# Enhanced Osteogenesis by Reduced Graphene Oxide/Hydroxyapatite Nanocomposites

**DOI:** 10.1038/srep18833

**Published:** 2015-12-21

**Authors:** Jong Ho Lee, Yong Cheol Shin, Sang-Min Lee, Oh Seong Jin, Seok Hee Kang, Suck Won Hong, Chang-Mo Jeong, Jung Bo Huh, Dong-Wook Han

**Affiliations:** 1 Department of Optics and Mechatronics Engineering, BK21+ Nano-Integrated Cogno-Mechatronics Engineering, College of Nanoscience & Nanotechnology, Pusan National University, Busan 609-735, South Korea; 2 Department of Prosthodontics, Pusan National University Dental Hospital, Dental Research Institute, School of Dentistry, Pusan National University, Yangsan 626-770, South Korea

## Abstract

Recently, graphene-based nanomaterials, in the form of two dimensional substrates or three dimensional foams, have attracted considerable attention as bioactive scaffolds to promote the differentiation of various stem cells towards specific lineages. On the other hand, the potential advantages of using graphene-based hybrid composites directly as factors inducing cellular differentiation as well as tissue regeneration are unclear. This study examined whether nanocomposites of reduced graphene oxide (rGO) and hydroxyapatite (HAp) (rGO/HAp NCs) could enhance the osteogenesis of MC3T3-E1 preosteoblasts and promote new bone formation. When combined with HAp, rGO synergistically promoted the spontaneous osteodifferentiation of MC3T3-E1 cells without hindering their proliferation. This enhanced osteogenesis was corroborated from determination of alkaline phosphatase activity as early stage markers of osteodifferentiation and mineralization of calcium and phosphate as late stage markers. Immunoblot analysis showed that rGO/HAp NCs increase the expression levels of osteopontin and osteocalcin significantly. Furthermore, rGO/HAp grafts were found to significantly enhance new bone formation in full-thickness calvarial defects without inflammatory responses. These results suggest that rGO/HAp NCs can be exploited to craft a range of strategies for the development of novel dental and orthopedic bone grafts to accelerate bone regeneration because these graphene-based composite materials have potentials to stimulate osteogenesis.

Calcium phosphates, such as hydroxyapatite (HAp) and β-tricalcium phosphate (β-TCP), which are known for their excellent biocompatibility and osteoconductivity, are used widely as clinically available bone substitutes^1^,^2^,^3^. In particular, HAp has been used for a long time in the oromaxillofacial field in areas, such as bone grafts, regeneration of defect areas and periodontal regeneration^4^. Owing to the very low absorption rate of HAp and its trabecular structure that helps introduce blood cells and bone cells, new bone deposition can be accelerated. This means that the material has the features of a scaffold with excellent biocompatibility^5^,^6^. On the other hand, according to a series of experimental studies, the proliferation and differentiation of osteoblasts was very low in the presence of HAp compared to the other bone substitutes^7^. To overcome this limitation, trials have been conducted to combine the osteoconductive scaffold with an osteoinductive protein to enhance the bone regeneration performance. Among the various osteoinductive proteins, bone morphogenetic protein-2 (BMP-2) can differentiate mesenchymal stem cells (MSCs) and preosteoblasts into osteoblasts, and promote the immigration of osteoblastic cells^8^. Despite this, the *in vivo* applications of BMP-2 have not been shown to significantly improve bone regeneration^9^,^10^. This poor *in vivo* result has been attributed to the rapid degradation of BMP-2 by proteinases; therefore, it was suggested that BMP-2 must be administered in more than milligram quantities^11^. A high concentration of BMP-2, however, can cause unwanted systemic abnormalities as well as local side effects, such as ectopic bone formation, osteoclast activation, cyst-like bone void formation and soft-tissue swelling^12^,^13^. HAp exhibits poor mechanical properties, such as intrinsic brittleness, low fracture toughness and low wear resistance. To improve mechanical and biological properties of HAp, most research has been implemented to combine it with other materials, e.g. polymers and carbon nanomaterials, for morphological and functional modifications^14^,^15^. In particular, the mechanical performance and biocompatibility of HAp had been improved significantly by reinforcement with reduced graphene oxide (rGO)^16^.

Over the last decade, graphene-family nanomaterials have been explored increasingly for biomedical applications including drug delivery carriers, imaging agents, biosensors and tissue engineering scaffolds owing to their exceptional physicochemical, optical, electrical and mechanical properties^17^,^18^,^19^,^20^. In particular, the potential of graphene and its derivatives has attracted significant attention as planar culture platforms or porous scaffolds for the differentiation of various types of SCs towards neurogenic^21^,^22^,^23^, osteogenic^24^,^25^, chondrogenic^26^, myogenic^27^, and adipogenic lineages^28^. On the other hand, the *in vivo* bioactive potential of graphene and its related materials remain to be explored. Most studies regarding graphene-related nanomaterials has concentrated on their toxicity, namely, if they are toxic or not *in vitro* and even *in vivo*^29^,^30^,^31^. Many of these studies have suggested that a high concentration of graphene does induce toxicity. A few studies have revealed genotoxicity at a lower concentration. Fewer studies have evaluated the use of graphene to prepare scaffolds for tissue engineering. These have typically involved assessing the cell response *in vitro* and have reported improved cell response. Nevertheless, the duration of these *in vitro* studies was insufficient for the full degradation of the polymer scaffolds and it was difficult to mimic the physiological situation^32^. The long term effects and associated risks, if any, of using graphene in tissue scaffolds *in vivo* are unclear and will require a more thorough assessment prior to its clinical use^33^. On the other hand, the two-dimensional nature of graphene makes it difficult to extend its applications beyond planar tissue cultures. To date, hybrid composites, composed of HAp and graphene derivatives, including GO and rGO, have been examined extensively in the context of osteogenesis in an *in vitro* cell culture model^16^,^34^,^35^; however, there are no reports of an evaluation of their osteogenic potency in an *in vivo* animal study, even though the direct combination of HAp with graphene is not novel. This study explored the potential of rGO/HAp nanocomposites (rGO/HAp NCs) to enhance osteogenesis both *in vitro* and *in vivo*.

## Materials and Methods

### Preparation of rGO nanosheets (NSs)

The GO was prepared from graphite according to the modified Hummers and Offeman method^36^. The small quantity of expandable graphite (Asbury Carbon, Grade 1721) as a source material was put in a glass beaker (500 mL) and heated using a microwave oven for about 10 sec. The volume of graphite was expanded about 150 times compared to its original volume. Concentrated H_2_SO_4_ (50 mM) was transferred into a glass flask (250 mL) containing a rotating magnetic stirrer and cooled in a cold water bath to 0 °C for the acid treatment process. Subsequently, the 2 g expanded graphite was slowly added to the flask and the 6 g KMnO_4_ was slowly added into the mixture to make a suspension. The suspension was heated for approximately 2 h at 35 °C under stirring, and then the de-ionized water (DIW) was added gradually to mixture, during which temperature was maintained less than 70 °C. To remove the KMnO_4_, H_2_O_2_ (30 wt.%) was added slowly. Vigorous bubbles were observed and the suspension color changed from dense brown to brilliant yellow. The suspension was several times filtered and diluted with DIW to completely remove acids until the dispersion became pH 6. Finally, the resulting suspension was dried over 12 h and the GO NSs were prepared. For the preparation of rGO NSs^37^, the prepared GO suspension (1 g in 1 L DIW) was sonicated for 2 h, and then the 10 mL hydrazine hydrate (N_2_H_4_ · H_2_O) was added to the GO suspension. The reaction was conducted at 100 °C for 24 h. Then, the suspension was several times filtered and washed with ethanol-water solution. Finally, after drying under vacuum condition at 80 °C for 12 h, the rGO NSs were prepared.

### Preparation and characterizations of rGO/HAp NCs

The HAp MPs were prepared using water-soluble calcium phosphate powder, HAp (HAp Tech Inc., Busan, Korea). Our previous study showed that the prepared HAp powder was analogous to the natural HAp, which was corroborated by X-ray diffraction (XRD) analysis, inductively coupled plasma-optical emission spectroscopy and energy dispersive spectroscopy^38^. To prepare rGO/HAp NCs, as-prepared rGO in DIW was sonicated for 2 h, and then mixed with a HAp MPs suspended in DIW with the weight ratio of 1:1. Colloidal dispersions of rGO NSs and HAp MPs were vigorously mixed using a vortex for 10 min and slowly air-dried at room temperature (RT) overnight, which results in the rGO/HAp NCs. The morphological structure of the rGO/Hap NCs was observed under a field emission scanning electron microscopy (FESEM, Hitachi S-4700, Hitachi, Tokyo, Japan) with an accelerating voltage at 5 kV. The mean particle size and Raman spectrum of the rGO/HAp NCs were measured by zetasizer (Malvern Instruments, Nano ZS, Worcestershire, UK) and Raman spectrometer (Micro Raman PL Mapping System, Dongwoo Optron Co., Ltd, Kwangju-si, Korea), respectively. To evaluate the crystalline structure of the rGO/HAp NCs, the XRD patterns were obtained by an X-ray dffractometer (Empyrean series 2, PANalytical, Almelo, Netherlands) with Cu-Kα radiation (λ = 0.154 nm) at 40 kV and 30 mA. The rGO NSs, HAp MPs and rGO/HAp NCs were scanned in the 2θ range of 10–60° at RT in the continuous scan mode (scan rate of 2θ = 2° min^−1^).

### Cell proliferation assay

A murine preosteoblastic cell line (MC3T3-E1 cells from C57BL/6 mouse calvaria) was purchased from the American Type Culture Collection (CRL-2593™; ATCC, Rockville, MD). The MC3T3-E1 cell is an established cell line as an osteoblast model that has been used for investigating the osteogenesis and bone formation^39^,^40^. The cells were maintained routinely in complete α-Minimum Essential Medium [basal media (BM), Sigma-Aldrich Co., St Louis, MO] supplemented with 10% fetal bovine serum (Sigma-Aldrich Co.) and 1% antibiotic antimycotic solution (Sigma-Aldrich Co.) in a humid incubator with 5% CO_2_ at 37 °C. To evaluate the proliferation of MC3T3-E1 cells, a cell counting kit-8 (CCK-8) assay (Dojindo Laboratories, Kumamoto, Japan) was carried out according to the manufacturer’s instruction. Briefly, the MC3T3-E1 cells were seeded in a 96-well plate at a concentration of 1 × 10^4^ cells mL^−1^ and cultured in the media at 37 °C in a 5% CO_2_ environment. Immediately after seeding, the cells were cultured in BM with colloidal dispersions of HAp MPs, rGO NSs or rGO/Hap NCs at 10 μg mL^−1^. Then, the cells were plated for 8–10 min and incubated as monolayers. The cells were cultured for 1, 7, 14, and 21 days, followed by two times washing with 1× Dulbecco’s phosphate-buffered saline (DPBS, Sigma-Aldrich Co.). The cells were then incubated with CCK-8 for 4 h in the dark at 37 °C. The effects of HAp MPs, rGO NSs or rGO/HAp NCs on cell proliferation were examined by measuring the absorbance at 450 nm with a SpectraMax^®^ 340 plate reader (Molecular Devices, Sunnyvale, CA). The proliferation of MC3T3-E1 cell was obtained by calculating the percentage of the optical density value in the cells (cultured with particles or composites at each time point) with respect to the optical density value of control at 1 day.

### Alkaline phosphatase (ALP) activity assay

The MC3T3-E1 cells were seeded in a 96-well plate at a concentration of 1 × 10^4^ cells mL^−1^ and cultured with colloidal dispersions of HAp MPs, rGO NSs or rGO/Hap NCs at 10 μg mL^−1^ for 1 to 21 days in BM at 37 °C in a 5% CO_2_ environment. The cells were then two times washed in DPBS and immersed in 0.1% Triton X-100 (Sigma-Aldrich Co.) in Tris-buffered saline (pH 7.5, Bioworld, Dublin, OH) for 10 min. The lysates were centrifuged at 13,000 × g for 3 min at 4 °C, and the supernatants were mixed with ALP assay working solution containing 1 mM ρ-nitrophenyl phosphate following manufacturer’s instruction (Alkaline Phosphatase Assay Kit, Abcam, Cambridge, MA). The ALP activity was expressed as the ρ-nitrophenol formation (μmol) divided by volume of sample (mL) and the reaction time (min).

### Alizarin red S (ARS) staining

ARS staining was performed to detect extracellular calcium deposits generated by MC3T3-E1 cells. The MC3T3-E1 cells were seeded in a 6-well plate at a concentration of 2 × 10^5^ cells mL^−1^ and cultured with colloidal dispersions of HAp MPs, rGO NSs or rGO/Hap NCs at 10 μg mL^−1^ for 1 to 21 days in BM at 37 °C in a 5% CO_2_ environment. The cells then were two times washed in DPBS and fixed in 2% paraformaldehyde (Sigma-Aldrich Co.) for 10 min. Cells then were stained for 20 min with ARS solution (pH 4.2, 40 mM, Sigma-Aldrich Co.) at RT. The stained plates were imaged using an inverted Leica DMIL microscope (Leica Microsystems, Wetzlar, Germany) and a digital camera (Olympus, Tokyo, Japan). The mineralization-positive cells were specifically stained red. To quantify the ARS staining, the stained cells were destained with a 10% acetic acid solution for 30 min, followed by neutralization in ammonium hydroxide solution. The absorbance was read in an ELISA reader at 405 nm.

### Von Kossa staining

Von Kossa staining is widely used together with ARS staining to monitor the mineralized bone nodules of the differentiated osteoblasts. Von Kossa staining is nonspecific for calcium ion, but it is positive for carbonate or phosphate deposits. The mineralized nodules are stained a brownish-blackish color. The MC3T3-E1 cells were seeded in a 6-well plate at a concentration of 2 × 10^5^ cells mL^−1^ and cultured with colloidal dispersions of Hap MPs, rGO NSs or rGO/Hap NCs at 10 μg mL^−1^ for 1 to 28 days in BM at 37 °C in a 5% CO_2_ environment. The cells then were two times washed in DPBS and fixed with 4% formaldehyde in sodium phosphate buffer for 10 min. After fixation, cells were then stained with a 5% silver nitrate solution under UV light for 30 min and three times washed in DIW. The staining was developed with aqueous 5% sodium carbonate (Na_2_CO_3_) in 25% formalin for over 5 min for mineralized bone nodules staining. Any residual silver nitrate solution were washed away by three times rinsing the culture dish with DIW and the cells were neutralized in 5% sodium thiosulfate for 2 min. Then, the cells were air-dried and imaged using a digital camera.

### Western blotting

The MC3T3-E1 cells were cultured with HAp MPs, rGO NSs or rGO/Hap NCs at 10 μg mL^−1^ for 21 days and two times washed in cold DPBS. After washing, ice-cold radioimmunoprecipitation lysis buffer (Santa Cruz Biotechnology, Santa Cruz, CA) was added for 5 min. The lysates were collected by centrifugation at 14,000 × g for 20 min at 4 °C. Thereafter, proteins in the cell lysates were extracted, and the total protein content was assessed with a bicinchoninic acid protein assay kit (Pierce, Rockford, IL) following manufacturer’s instruction. Western blotting analysis was carried out to evaluate the protein expression level. The equal amount of protein (40 μg) was subjected to electrophoresis on a 4–20% sodium dodecyl sulfate-polyacrylamide gels (Daiichi Pure Chemicals, Tokyo, Japan) at 30 mA for 1 h and electrotransferred to polyvinylidenefluoride membranes at 35 mA for 50 min. The protein-transferred membranes were blocked with Blocking One Buffer (Nacalai Tesque, Kyoto, Japan) for 1 h at RT and probed using rabbit anti-osteopontin (OPN) monoclonal and mouse anti-osteocalcin (OCN) monoclonal antibodies (Abcam) at 1:1000 and 1:250 dilutions, respectively. The expression of glyceraldehyde-3-phosphate dehydrogenase (GAPDH) was regarded as an internal control. The membranes were reacted with goat anti-rabbit (Santa Cruz Biotechnology) or goat anti-mouse (Amersham Biosciences, Buckinghamshire, UK) secondary antibodies conjugated with horseradish peroxidase at 1:2,000 dilutions for 1 h at RT. The expression of proteins was determined by a Chemilumi-one chemiluminescent kit (Nacalai Tesque) and X-ray film (Fujifilm, Tokyo, Japan). Densitometric analysis was performed using a Scion Image software (Scion Co., Frederick, MD). Expression density was determined by calculating the percentage of the band density in the cells (cultured with particles or composites) with respect to the band density of control.

### Preparation of bone grafts

For the animal study, two types of bone grafts were prepared, as shown in Figure S7; one was a HAp graft and the other was an rGO/HAp graft. The HAp grafts were prepared by mixing 1 g of HAp MPs and 0.4 mL of DPBS, which were made into a paste, and then contained in a 1 mL syringe and kept at 4 °C prior to use. In the case of the rGO/HAp grafts, 1 g of HAp MPs was blended gently with 0.4 mL of a 500 μg mL^−1^ rGO solution in DPBS, where the HAp to rGO weight ratio was 5,000:1. The morphology and elemental distribution of the rGO/HAp NCs constituting the grafts were examined by FESEM (Hitachi S-4700) and energy dispersive X-ray (EDX) spectroscopy, respectively.

### Animals and surgery

Six 12- to 13-week-old male New Zealand white rabbits (3.3–3.5 kg in weight) were housed in a light- and temperature-controlled environment and provided with food and water ad libitum. All animal experiments were approved by the Ethics Committee on Animal Experimentation at Chonnam National University and performed in accordance with the Animal Care and Use Committee guidelines of Chonnam National University (CNU IACUC-YB-2013-31). The rabbits were anesthetized by an intraperitoneal injection with a combination of 75 mg/kg ketamine HCl (Huons Co., Ltd., Seongnam-si, Korea) and 10 mg/kg xylazine hydrochloride (Rompun^®^; Bayer AG, Leverkusen, Germany). The dorsal section of the cranium was shaved and prepared aseptically for surgery, and a sagittal incision, approximately 20 mm in length, was opened over the scalp of the animal. The periosteum was removed and a full-thickness bone defect (6 mm in diameter and 2.5 mm in depth) was trephined in the center of each parietal bone using a slow-speed dental drill (Marathon 3, Saeyang Co., Daegu, Korea) without damaging the dura (four grafts per one calvarium). The bone defects were implanted randomly with either HAp or rGO/HAp grafts and were left empty as the non-treated control (Figure S8). After implanting the grafting materials, a biocellulose membrane (Jadam Co., Jeju, Korea) was covered to prevent the loss of grafts. The graft amount was pre-determined based on the defect volume, and all defect sites received the same graft volume. The mean volume of the prepared grafts in each defect site was 70.65 mm^3^. Each experimental group was comprised of eight samples and the total number of prepared defects was 24. The animals were allowed to recover for 4 weeks after surgery, at which time, they were sacrificed by CO_2_ inhalation. To collect the implanted grafts, the skin was dissected and the defect sites were removed along with the surrounding bone. The biopsied specimens were fixed and prepared for micro-CT analysis and histomorphometric analysis.

### Histopathology and histomorphometric analysis

The tissue specimens of the rabbit calvarias were decalcified using 14% ethylenediaminetetraacetic acid (EDTA, Sigma-Aldrich Co.) and rapid acid decalcification reagents. The specimens were then embedded in paraffin and sectioned at 5 μm in the centers of calvarial defects. The two most central sections in each block were selected and stained with hematoxylin-eosin (HE) and Masson’s trichrome (MT). The prepared histology slides were observed by optical microscopy (BX50, Olympus Optical Co.), and the images were digitally captured. To obtain the areas of new bone and of the residual graft materials in the images, computer-assisted histometric measurements were taken using an image analysis program (Image-Pro Plus, Media Cybernetics, Silver Spring, MD). The percentage ratios of the new bone and residual grafts in all defect areas were calculated using the equation presented in Figure S9. The total neo-tissue (augmented) area rate means the percentage ratio of newly formed bone (n) plus the residual HAp or rGO-coated HAp grafts (r) plus fatty marrow and fibrovascular tissues (f) to the total region of the defect area. New bone density was obtained as the percentage ratio of newly formed bone to the total neo-tissue area (n + r + f).

### Statistical analysis

All quantitative data were expressed as means ± standard deviation (SD) from duplicate experiments carried out in three independent cultures for each *in vitro* experiment. The data were tested for the homogeneity of variances using a Levene test, prior to statistical analysis. Multiple comparisons to detect the effects of rGO/HAp NCs on the proliferation, ALP activity, mineralized bone nodules and the expression of osteogenic proteins in MC3T3-E1 cells was performed with analysis of variance (ANOVA), followed by a Bonferroni test when variances were homogeneous or a Tamhane test when variances were not. A value of *p* < 0.05 was considered statistically significant. The SPSS package (version 18.0, IBM Co., Chicago, IL) was used for statistical analysis of the animal study. A Kruskal-Wallis test was used to determine the differences in the micro-CT and histomorphometric measurements between the experimental groups 4 weeks after surgery. Statistical significance was accepted for a value of *p* < 0.05.

## Results and Discussion

### Characterizations of rGO-coated HAp composites

**Figure 1:**
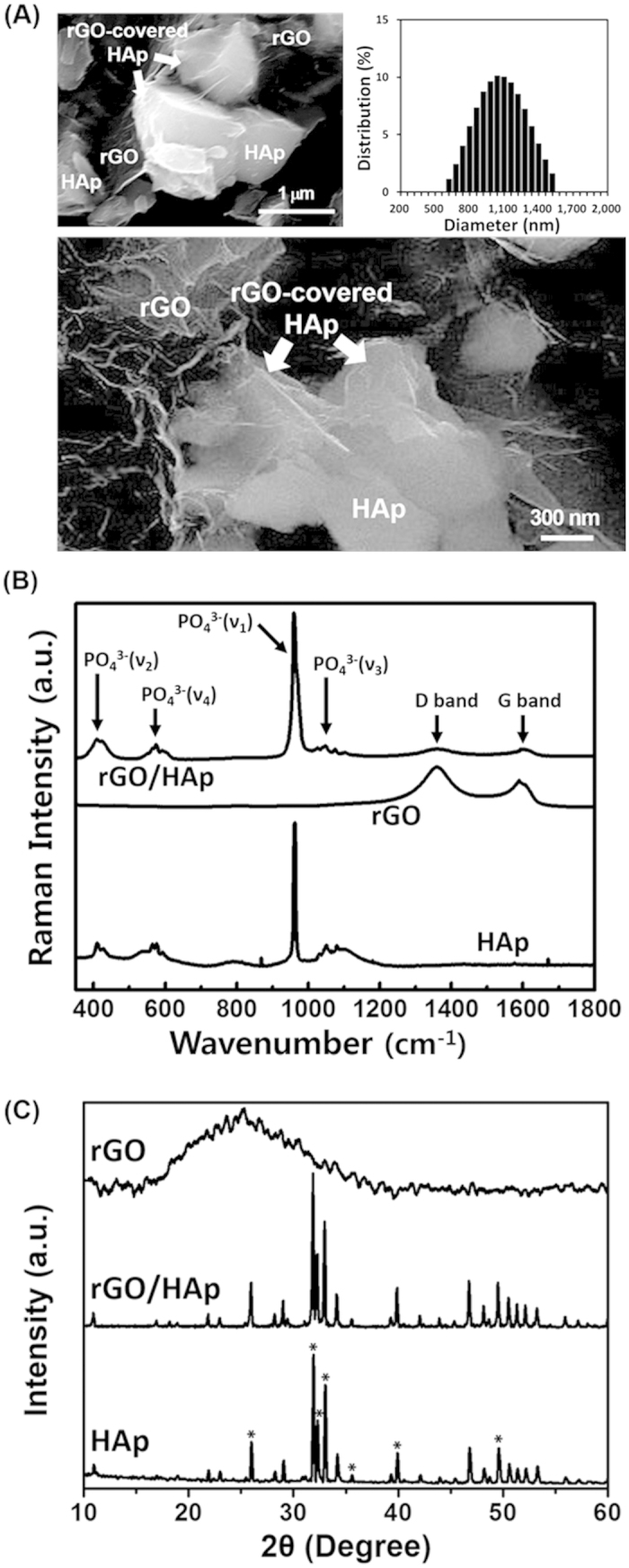
Physicochemical characteristics of the rGO/HAp NCs. (**A**) FESEM images of the rGO/HAp NCs show that the morphology of the HAp MPs was irregular-shaped granules with a mean particle size of 960 ± 300 nm as well as that HAp MPs were partly covered and interconnected by an network of rGO NSs. (**B**) Raman spectra of HAp MPs, rGO NSs and rGO/HAp NCs indicate that the rGO NSs wrap the surface of HAp MPs. (**C**) XRD patterns of HAp MPs, rGO NSs and rGO/HAp NCs reveal that the diffraction peaks of HAp MPs were well in accordance with the standard pattern of HAp (JCPDS card No. 09–0432) and the XRD pattern of rGO/HAp NCs was highly similar to that of HAp MPs.

FESEM showed that the HAp microparticles (MPs) had an irregular-shaped granular morphology with a mean size of 960 ± 300 nm, and that HAp MPs were partially covered and connected by a network of rGO NSs (Fig. 1A). Previous study revealed GO/graphene–HAp hybrid composites can not only support high viability of osteoblast cells but can also provide a comparable microenvironment to that found *in vivo*^41^. The surface potential of rGO is negatively charged due to the hydroxyl and carboxyl groups on their surface, whereas HAp MPs are positively charged because of their calcium moieties. Therefore, it is considered that rGO/HAp NCs are formed through electrostatic interactions between HAp MPs and rGO NSs. In addition, a hydrogen-bonding interaction between the hydroxyl groups of HAp MPs and the oxygen-containing functional groups of rGO NSs such as carboxyl, hydroxyl, carbonyl, and epoxy groups may also provide a good adhesion between HAp MPs and rGO NSs^42^. Raman spectroscopy was performed to investigate the composition of the rGO/HAp NCs. In the Raman spectrum of the rGO/HAp NCs (Fig. 1B), several characteristic peaks were observed at 428, 576, 962, and 1049 cm^−1^, which represented the phosphate groups in HAp MPs^43^. Additionally, the characteristic bands of rGO NSs were found at 1,360 and 1600 cm^−1^, which were assigned to the D and G band of rGO NSs, respectively^44^,^45^. There was no shift in Raman spectra of HAp MPs and rGO NSs in rGO/HAp NCs, suggesting that the rGO/HAp NCs were successfully prepared, as shown in Fig. 1A. The XRD pattern of rGO NSs exhibits a broad diffraction peak at 25.3°, corresponding to the interlayer spacing (*d*-spacing) of 0.351 nm, which was calculated by the Bragg equation (Fig. 1C). It should be noted that the *d*-spacing of rGO (0.351 nm) is smaller than that of pure GO (~0.83 nm)^46^,^47^, due to the removal of oxygen-containing functional groups^48^. The XRD pattern of HAp shows noticeable peaks at 25.9° and 31.9°, corresponding to the (002) and (211) planes, respectively. In addition, characteristic peaks were observed at 32.3°, 33.0°, 35.6°, 39.9°, and 49.6°, corresponding to the (112), (300), (202), (310), and (213) planes, respectively. The diffraction peaks of HAp are well in accordance with the standard pattern of HAp (JCPDS card No. 09–0432), suggesting that the HAp MPs have a hexagonal crystalline structure with high purity^49^. On the other hand, the XRD pattern of rGO/HAp NC is highly similar to that of HAp. This can be attributed to the fact that the diffraction peak of rGO is relatively weak and broad compared to that of HAp due to the amorphous nature of rGO^50^. However, according to the XRD patterns together with FESEM observation and Raman spectroscopy, it was shown that the rGO and HAp retained their structure in the rGO/HAp NCs.

### Cytotoxicity of rGO/HAp NCs

We examined the influence of HAp MPs, rGO NSs and rGO/HAp NCs on the MC3T3-E1 cells by measuring their metabolic activity using CCK-8 assay to assess their cytotoxicity. The amount of the formazan dye, the metabolic reaction products obtained in the CCK-8 assay, was determined because the number of viable cells was fairly proportional to them. As shown in Figure S1, the viability of the MC3T3-E1 cells was apparently decreased with increasing concentrations of HAp MPs, rGO NSs and rGO/HAp NCs. The HAp MPs have been shown to cause significant (*p* < 0.05) reduction of relative cell viability from 15.6 μg mL^−1^ and to induce the loss of more than half of the cell viability at 125 μg mL^−1^, having IC_50_ value of 67 μg mL^−1^ (Figure S1A). At higher concentrations than 31.3 μg mL^−1^, HAp MPs are considered to exhibit toxicity against cells due to their intrinsic characteristics, e.g. alkalinity. Colloidal dispersions of HAp MPs at 10 and 100 μg mL^−1^ increased the pH values of the culture media up to 7.97 and 8.20 (from 7.32), respectively. This result is supported by evidence showing that the HAp nanoparticles significantly inhibited the growth of MC3T3-E1 cells, even at 10 μg mL^−1^ concentration^51^. Considering only the cytotoxicity *in vitro*, it appears that HAp MPs are able to be safely applied to bone grafts and fillers at least at lower concentrations than 10 μg mL^−1^. On the other hand, rGO NSs and rGO/HAp NCs at lower concentrations than 31.3 μg mL^−1^ did not show any significant reduction in the cell viability, and their IC_50_ values were estimated to be about 875 and 1091 μg mL^−1^, respectively (Figure S1B and S1C). In particular, the cytotoxicity of the HAp MPs was mitigated appreciably by coating them with rGO NSs. Recently, it was reported that neither significant cellular uptake nor apparent cytotoxicity of GO and graphene were observed in macrophages and epithelial cells at relatively low concentrations (<50 μg mL^−1^), whereas higher concentrations triggered oxidative stress that decreased slightly the cell viability^52^,^53^.

### Effects of rGO/HAp NCs on proliferation of MC3T3-E1 cells

**Figure 2:**
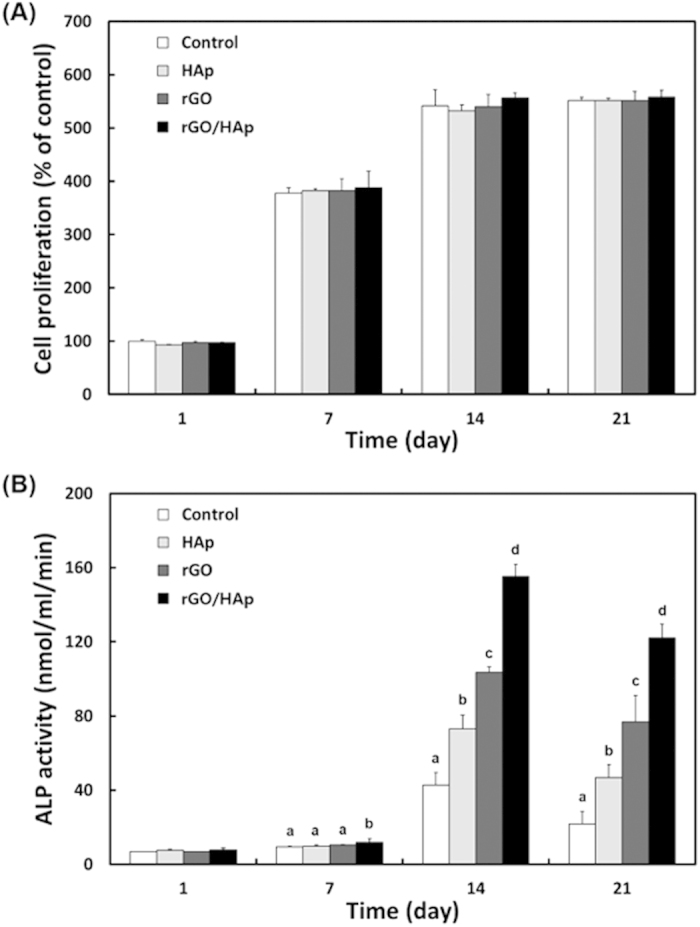
Proliferation and ALP activity of MC3T3-E1 cells incubated with a colloidal dispersion of HAp MPs, rGO NSs or rGO/HAp NCs in BM. (**A**) During the incubation period (up to 21 d), the presence of rGO/HAp NCs resulted in no appreciable decrease in cell proliferation, compared to the non-treated control. (**B**) Incubation with rGO/HAp NCs for 7 to 21 d significantly (*p* < 0.05) induced ALP activity. The data is expressed as the mean ± SD based on at least duplicate observations from three independent experiments. The different letters in (**B**) denote the significant difference between the non-treated control and the cells incubated with particles or composites, *p* < 0.05.

The addition of HAp MPs, rGO NSs and rGO/HAp NCs to the BM did not cause any decrease in the proliferation of MC3T3-E1 cells compared with the non-treated control after 1 day of incubation (Fig. 2A). At 21 days of incubation, the proliferation patterns of the cells incubated with the particles or composites were found to be similar to that of the non-treated control. This result suggests that the cellular proliferation was not adversely affected by particles or composites initially exposed to the serum contained in the media. Although the cell type and culture condition as well as the treatment concentration were different from the present study, some contrasting results were reported showing that when cultured on GO-HAp coatings or treated with relatively higher concentrations of rGO/HAp composites, osteoblast-like cells, such as MG63 and hFOB 1.19 cells, showed better adhesion and proliferation than the cells cultured on uncoated surfaces or non-treated cells^16^,^34^. This conflicting phenomenon can be explained by the difference in the physicochemical properties, such as surface charge, the particle size and composition ratio of HAp-rGO NCs. When MC3T3-E1 cells were cultured with those particles or composites at the same concentration in osteogenic media (OM) with osteoinductive agents including β-glycerophosphate, dexamethasone and L-ascorbic acid^54^,^55^, the pattern of proliferation was at a similar level to that observed without those agents (Figure S2A). In the present study, firstly the cells were seeded and then the cell suspension was cultured in BM with colloidal dispersions of HAp MPs, rGO NSs or rGO/HAp NCs until they were cultured as monolayers. This method has advantages in that the osteoblasts can be exposed primarily to rGO/HAp NCs in 3D cultures than as 2D adherent monolayer, and the effectiveness of interaction between cells and those NCs increased, then, leading to facilitate intracellular signaling and successive osteoactivity in the osteoblastic cells^56^,^57^. On the other hand, macroporous 3D tissue scaffolds composed of poly(*ε*-caprolactone) incorporated with strontium-decorated rGO hybrid nanoparticles enhanced the proliferation and differentiation of MC3T3-E1 cells significantly^32^.

### Effects of rGO/HAp NCs on ALP activity of MC3T3-E1 cells

There was no consistent correlation between the cellular ALP activity and cell proliferation (Fig. 2B), which might be owing to the instantaneous ALP secretion into the culture media because it is a secreted enzyme. The proliferation of osteoblasts greatly increased for 7 ~ 14 days, then begin to secrete extracellular matrix proteins and express early markers for osteodifferentiation, including ALP, after 7 days^58^. When the MC3T3-E1 cells were cultured with colloidal dispersions of HAp MPs or rGO NSs in BM in the absence of any osteogenic agents, remarkable changes were not observed until day 7. The cells cultured with 10 μg mL^−1^ rGO/HAp NCs for 14 days exhibited significantly (*p* < 0.05) increased ALP activity compared to those cultured with HAp MPs or rGO NSs only. This indicates that rGO/HAp NCs can enhance the early osteogenic differentiation marker without any osteogenic agents. Moreover, culture with rGO/HAp NCs was more potent than culture with HAp MPs or rGO NSs only in enhancing ALP activity until day 21, suggesting that HAp MPs and rGO NSs synergistically act in osteodifferentiation. Some studies reported that HAp-GO (or -rGO) composites elevated the ALP activity of MG63 and hFOB 1.19 cells substantially^59^,^60^. Interestingly, rGO NSs alone showed superior potential in inducing ALP activity to HAp MPs alone. On the other hand, when MC3T3-E1 cells were cultured in OM for 7 days, the cells showed remarkably higher ALP activity (>40 nmol mL^−1^ min^−1^) than the cells cultured in BM (~10 nmol mL^−1^ min^−1^), regardless of the addition of particles or composites. At 21 days, the ALP activities of MC3T3-E1 cells cultured in OM with rGO NSs only and rGO/HAp NCs at the same concentration as BM reached approximately 5.0 and 3.4 times their values in BM, respectively (Figure S2B).

### Effects of rGO/HAp NCs on calcium deposition and matrix mineralization in MC3T3-E1 cells

**Figure 3:**
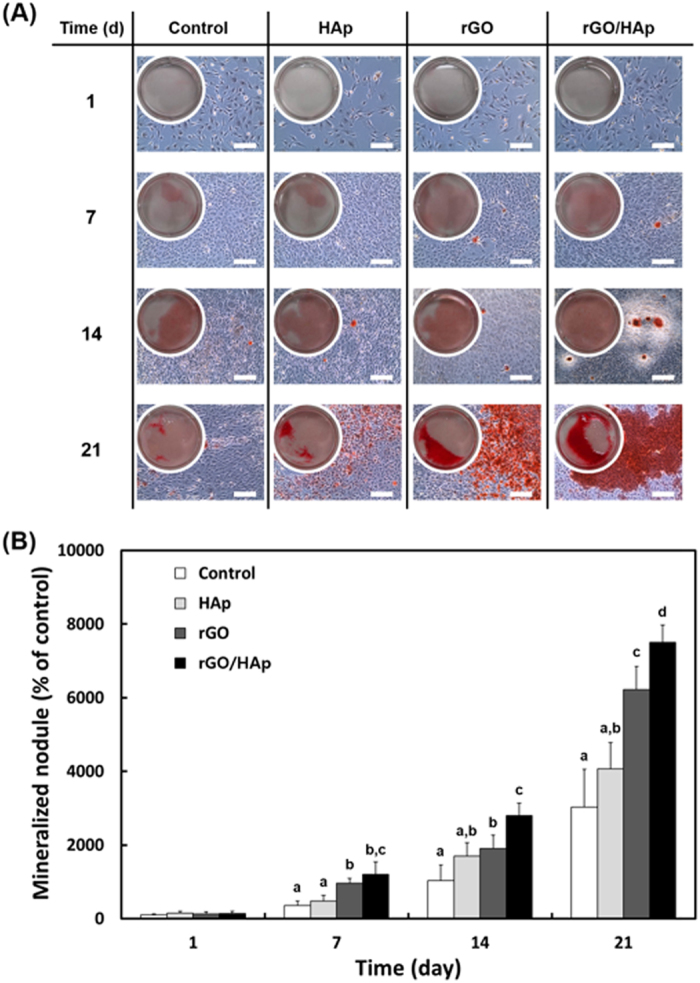
The ARS stain and its corresponding extract in MC3T3-E1 cells incubated with a colloidal dispersion of HAp MPs, rGO NSs or rGO/HAp NCs in BM. (**A**) Increased calcium deposits by rGO/HAp NCs were not related to the cell number (scale bars = 200 μm). There was a notable formation of calcium deposits by rGO/HAp NCs from 14 d. (**B**) The dissolved ARS extracted from the staining plates confirmed that the rGO/HAp NCs significantly (*p* < 0.05) increased extracellular calcium deposition in the cells. The data is expressed as the mean ± SD based on at least duplicate observations from three independent experiments. The different letters in (**B**) denote the significant difference between the non-treated control and cells incubated with particles or composites, *p* < 0.05. All photographs shown in this figure are representative of six independent experiments with similar results.

The ARS stain images and their corresponding extractive substances revealed that rGO/Hap NCs at 10 μg mL^−1^ increased calcium deposits in MC3T3-E1 cells significantly (Fig. 3). The deposition of calcium phosphate in the extracellular matrix is indicative of osteogenesis and has been taken as a marker for bone regeneration. The bioactivity of a scaffold for bone tissue engineering has been widely assessed by examining the *in vitro* mineralization by osteoblasts cultured in the scaffold^32^. Increased extracellular calcium deposition by the rGO/HAp NCs were unrelated to the number of cells, as shown by the morphology using optical microscopy (Fig. 3A). Significant increase of calcium deposition by rGO/HAp NCs was observed between day 14 and day 21, suggesting that HAp MPs and rGO NSs synergistically enhanced deposition of calcium in MC3T3-E1 cells. Surprisingly, rGO NSs alone exhibited significantly (*p* < 0.05) greater calcium deposition than HAp MPs alone at 21 days. Moreover, the dissolved ARS extracted from the stained monolayer of cells also quantitatively confirmed the above qualitative calcium staining pattern (Fig. 3B). Calcium deposits in the extracellular matrix generated by the MC3T3-E1 cells cultured with rGO/HAp NCs were comparable to a fibril-like appearance in which calcium deposition in the matrix was related within collagen fibers. In contrast, ARS staining of the cells cultured in OM exhibited a dispersed granule-like formation of calcium deposits (Figure S3A). The ARS extracted from the cells incubated for 21 days in OM showed that rGO/HAp NCs induced higher calcium deposition than the non-treated controls and HAp MPs alone (Figure S3B). These results imply that rGO/HAp NCs can stimulate the late osteogenic differentiation marker without any osteogenic agents. A recent study showed that the differentiation potential of human adipose derived MSCs to osteoblasts did not change significantly when treated with 2D graphene nanostructures, including graphene nano-onions, GO nanoribbons and GO nanoplatelets at a low (10 μg mL^−1^) and high (50 μg mL^−1^) concentrations for 24 h^61^, which was completely different from the treatment with rGO-based composites.

**Figure 4:**
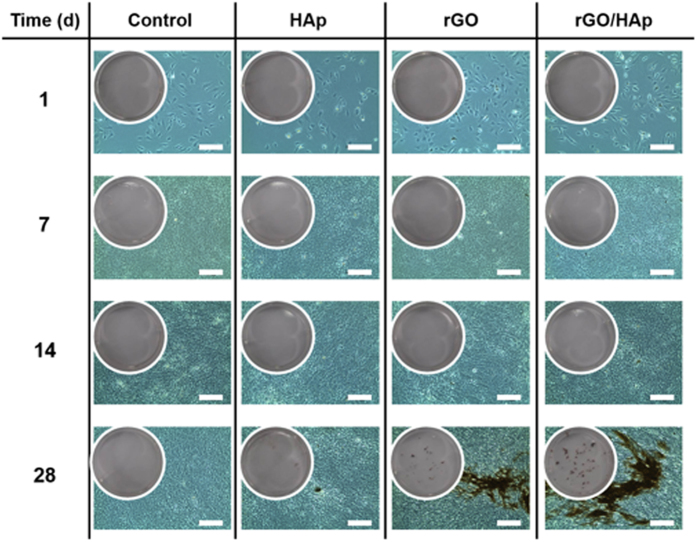
Image of von Kossa stain in MC3T3-E1 cells incubated with a colloidal dispersion of HAp MPs, rGO NSs or rGO/HAp NCs in BM. Dark brown colored nodular staining was observed at 28 d in cells incubated with rGO/HAp NCs (scale bars = 200 μm). There was little, if any, crystal formation in cells incubated with HAp MPs alone, whereas the cells incubated with rGO NSs alone exhibited strong positivity for von Kossa staining. All photographs shown in this figure are representative of six independent experiments with similar results.

The image of von Kossa staining showed that rGO/HAp NCs at 10 μg mL^−1^ induced significant osteodifferentiation with the concomitant generation of mineralized bone nodules by MC3T3-E1 cells (Fig. 4). The mineralized bone nodules stained dark brown were observed at day 28 in the MC3T3-E1 cells incubated with rGO/HAp NCs, while little crystal formation was observed in the cells cultured with Hap MPs alone (Fig. 4). Additionally, MC3T3-E1 cells cultured with rGO NSs alone were strongly positive to von Kossa staining, demonstrating the formation of mineralized nodules in the cells. On the contrary, in the control cultures without any treatment of particles or composites, von Kossa staining was negative. Crystal formation with dense brown mineralized nodules was observed at 28 days in the MC3T3-E1 cells incubated in OM, regardless of the particles or composites addition (Figure S4). These results indicate that rGO/Hap NCs may stimulate the later osteogenic differentiation marker in the absence of the addition of osteogenic agents^62^. ALP is an early stage marker of osteodifferentiation, and its activity decreases as the matrix matures. In contrast, calcium is a late stage marker of osteodifferentiation, and its activity increases as the matrix matures. Therefore, higher levels of ALP activity, combined with higher calcium deposits for rGO/HAp NCs at 10 μg mL^−1^ compared to the non-treated controls and HAp MPs alone, indicated that the matrix had matured completely. This assessment was corroborated further from von Kossa staining, showing the mineralized matrices by differentiated osteoblasts.

### Effects of rGO/HAp NCs on expressions of osteogenic proteins in MC3T3-E1 cells

**Figure 5:**
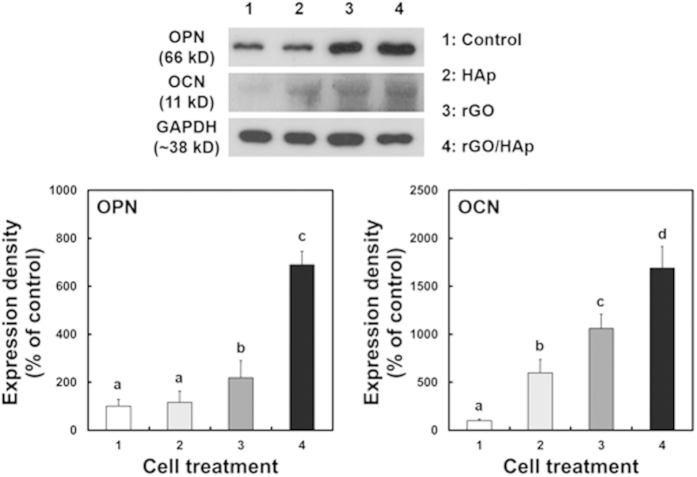
Immunoblotting for OPN and OCN expression in MC3T3-E1 cells incubated with a colloidal dispersion of HAp MPs, rGO NSs or rGO/HAp NCs in BM. After 21 d of incubation, the expression levels of both osteogenic proteins were increased significantly (*p* < 0.05) by rGO/HAp NCs. These results show good consistency with those of ARS and von Kossa staining, which are other markers for late osteogenic differentiation. The data is expressed as the mean ± SD based on at least duplicate observations from three independent experiments. The different letters denote the significant differences between the non-treated control and the cells incubated with particles or composites, *p* < 0.05.

The expression of the osteogenic proteins such as OPN and OCN as the markers of early and terminal osteoblast differentiation, respectively, were assessed by immunoblot analysis (Fig. 5). The expression levels of both proteins in MC3T3-E1 cells incubated in BM without any osteogenic agents for 21 days were increased significantly (*p* < 0.05) by 10 μg mL^−1^ rGO/HAp NCs. rGO NSs alone caused an appreciable increase in OPN and OCN expression as the matrix proteins for calcification, while Hap MPs alone could not markedly influence the OPN expression. In contrast, the non-treated cells exhibited little, if any, increase in the expression of both osteogenic proteins. These results are well in accordance with ARS and von Kossa staining results, which are the other late osteogenic differentiation markers, suggesting that rGO/HAp NCs stimulate the osteodifferentiation of MC3T3-E1 preosteoblasts. These findings were further supported by a recent study showing that the proliferation rate and differentiation of human osteoblast cells on the calcium silicate/rGO composites were improved remarkably^63^. Another study also reported that GO-calcium phosphate NCs have synergistic effects in accelerating the osteodifferentiation of MSCs by greatly increasing calcium deposition^64^, underscoring the need for such hybrid NCs for bone tissue engineering. The rGO/HAp NCs in the form of a colloidal dispersion added to the culture medium is believed to promote osteogenic differentiation and enhance the metabolic activity of osteoblasts.

### Inflammatory responses, micro-CT analysis and histological observations

The results of quantitative real time-polymerase chain reaction (qRT-PCR) analysis to determine the relative mRNA expression levels of interleukin 6 (IL-6) and tumor necrosis factor-α (TNF-α) showed that compared to normal tissue, no specific inflammatory responses were observed in the HAp grafts and rGO/HAp grafts at 4 weeks of surgery, but the mRNA expression levels of IL-6 and TNF-α were increased significantly (*p* < 0.05) in the non-treated control (Figure S5). These reduced local inflammation responses at the defects implanted with the rGO/HAp grafts are believed to subsequently enhance bone regeneration. When implanted into a critical-sized bone defect of rats, the hydrogels incorporating mixed triptolide, which is known as an immunosuppressive and anti-inflammatory agent, and BMP-2 had been shown to have a significantly smaller number of neutrophils, lymphocytes, macrophages or dendritic and mast cells infiltrated into the defect, as well as lower level of IL-6, TNF-α, and IL-10 expression than those incorporating BMP-2 without triptolide-micelles^65^. The total volume of newly formed bone within the region of interest (ROI) was measured by assigning a threshold for the total bone content (including trabecular and cortical ranges) and subtracting any contribution from the graft. Both HAp and rGO/HAp grafts showed substantially greater neo-tissue areas than the non-treated control (Figure S6A). The relative micro-CT values for new bone formation 4 weeks postoperatively were 11.68 ± 8.99, 609.30 ± 308.58 and 1157.83 ± 224.52 in the control, HAp grafts and rGO/HAp grafts, respectively (Figure S6B). A significant difference (*p* < 0.05) in new bone formation was observed between HAp grafts and rGO/HAp grafts.

**Figure 6:**
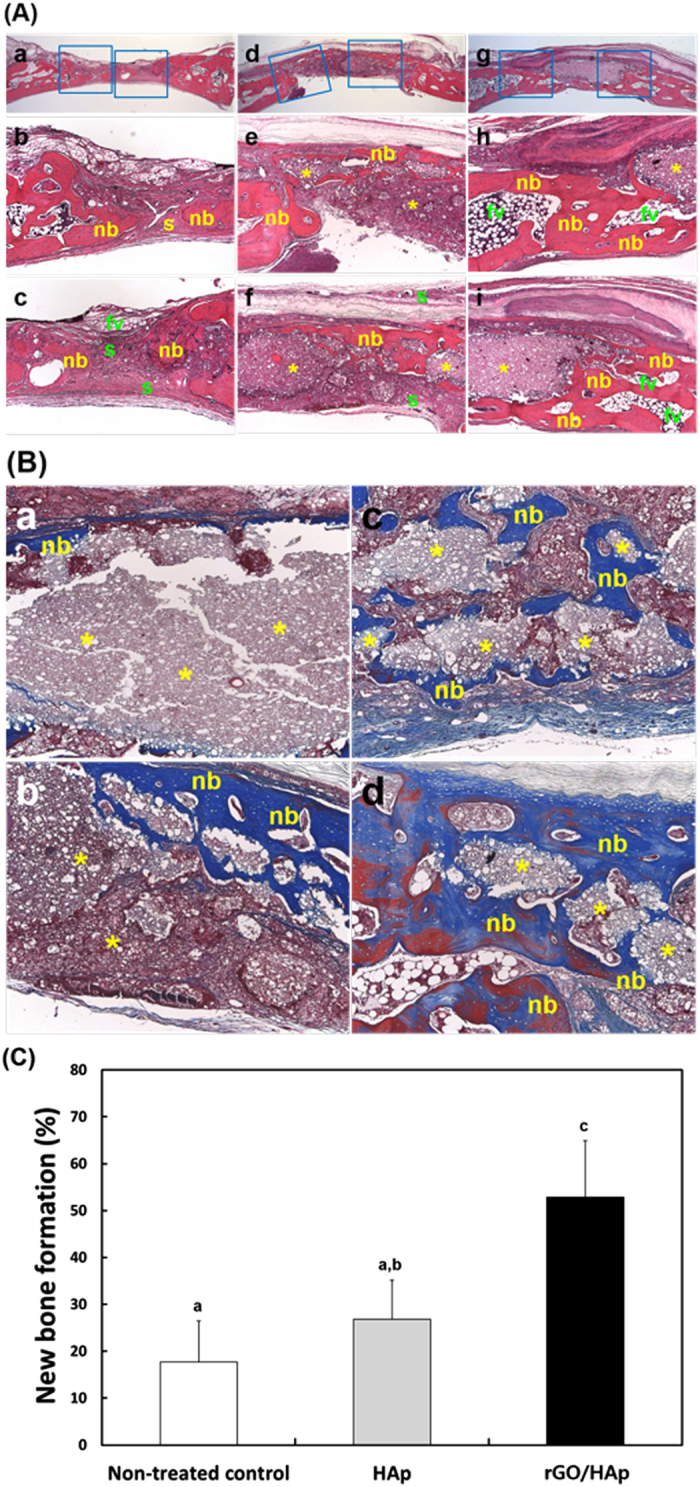
Histological observations. (**A**) Images from HE staining. In the non-treated control (**a**–**c**), the defects were filled with a thin, loose connective tissue with minimal new bone formation originating from the defect margins and fibrovascular tissues 4 weeks after surgery. In the defects treated with the HAp grafts (**d**–**f**), the defect sites were filled with dense connective tissue and small particles (**d**). Minimal new bone formation, fatty marrow and fibrovascular tissues were observed (**e**). Most of the HAp MPs did not appear to be resorbed (**f**). In the defect treated with rGO/HAp grafts (**g**–**i**), a larger number of residual HAp particles associated with a large number of giant cells and inflammatory round cells were present within loose, fibrous connective tissue at the defect site (**g**). In addition, some new bone formation adjacent to the defect margins was observed ((**h**,**i**)). Original magnifications: ×12.5 in (**a**,**d**,**g**) and ×50 in the others. (**B**) Images from MT staining. The quantity of newly formed bone in the rGO/HAp grafts was substantially higher than that in the HAp grafts, showing more advanced stages of remodeling and consolidation. Furthermore, bony tissue partially enveloped bone graft materials and new bones were observed in the blank spaces around the rGO/HAp grafts ((**a**,**b**) HAp grafts and (**c**,**d**) rGO/HAp grafts). Original magnifications: ×100. Symbols: nb, new bone; s, soft tissue; fv, fibrovascular tissue; *, graft materials. (**C**) New bone formation (%). The histometric analysis showed that the new bone density in rGO/HAp grafts was significantly greater (*p* < 0.05) than that in the other groups. The data is expressed as the mean ± SD based on at least duplicate observations from four independent specimens. The different letters in (**C**) denote a significant difference between the non-treated control and defects implanted with HAp and rGO/HAp grafts, *p* < 0.05.

In the non-treated control, the defects were filled with thin, loose connective tissue with minimal new bone formation originating from the defect margins and fibrovascular tissues 4 weeks after surgery (Fig. 6A(a–c)). In the defect treated with the HAp grafts, the defect sites were filled with dense connective tissue and small particles (Fig. 6A(d)). Minimal new bone formation, fatty marrow and fibrovascular tissues were observed (Fig. 6A(e)). Most of the HAp MPs did not appear to have been resorbed (Fig. 6A(f)). In the defect treated with rGO/HAp grafts, a larger number of residual HAp particles associated with a large number of giant cells and inflammatory round cells were present within the loose, fibrous connective tissue at the defect site (Fig. 6A(g)). In addition, some new bone formation adjacent to the defect margins was observed (Fig. 6A(h,i)). These results were confirmed further by MT staining. The quantity of the newly formed bone in the rGO/HAp grafts was substantially greater than that observed in the HAp grafts, showing more advanced stages of remodeling and consolidation. Furthermore, bony tissue partially enveloped the bone graft materials and new bone was found in blank spaces around the rGO/HAp grafts (Fig. 6B). Histometric analysis demonstrated that the mean (±SD) values for the new bone density (%) in the control, HAp grafts and rGO/HAp grafts were 17.66 (±8.81), 26.80 (±8.32) and 52.85 (±12.04), respectively (Fig. 6C). This result correlated well with that of the relative micro-CT values for new bone formation. The rGO/HAp grafts showed significantly greater (*p* < 0.05) new bone density than the other groups. On the other hand, there was no significant difference in the new bone density between the control and HAp grafts. Recently it was reported that GO-coated titanium implants can be used for the dual delivery of both BMP-2 and substance P, and that this delivery promotes bone formation in a mouse calvarial defect model^66^. Although not applied in combination with BMPs, the rGO/HAp graft in the form of a paste implanted into the calvarial defect is believed to promote osteogenesis. The aim of this study was to demonstrate the utility of developing hybrid NCs for bone regeneration and the advantages that it may offer. Our findings show that the rGO/Hap NCs can be successfully applied to prepare multifunctional composites with excellent biocompatibility and osteoinductivity. These graphene-based hybrid composites are more likely to find use in the next generation of nanobiomaterials for bone tissue engineering.

## Conclusions

The osteogenesis of MC3T3-E1 cells was stimulated spontaneously by rGO/HAp NCs without osteoinductive agents. Moreover, the osteogenic responses mediated by rGO/Hap NCs were more stimulated when the cells were incubated in the presence of osteogenic agents. These results might be explained by the fact that the cells were exposed primarily to rGO/Hap NCs in 3D cultures and the subsequent effectiveness of interaction between cells and those NCs was increased, then, leading to promoted intracellular signaling. The osteoinductive potential of rGO/HAp NCs observed in the *in vitro* cell culture model were also corroborated by the animal study showing that the rGO/HAp NCs were quite effective on new bone formation in a full-thickness calvarial defect. On the other hand, further comprehensive mechanisms, including intracellular signal transduction pathway, are still not fully understood and will require additional studies at a molecular level. Anyway, these results suggest that the rGO/HAp NCs will be promising candidate as a scaffold in bone regeneration, stimuli for the osteodifferentiation of osteoprogenitor cells and elements of dental devices, because of their effects on enhancing osteogenesis. Therefore, this work would be of paramount importance in the context of crafting strategies for the development of novel coating agents and bone fillers for dental implants based on graphene-based composites and for further controlled acceleration-required bone regeneration.

## Additional Information

**How to cite this article**: Lee, J. H. *et al.* Enhanced Osteogenesis by Reduced Graphene Oxide/Hydroxyapatite Nanocomposites. *Sci. Rep.* **5**, 18833; doi: 10.1038/srep18833 (2015).

## Supplementary Material

Supplementary Information
