# Supplementary material for: Enhanced Osteogenesis by Reduced Graphene Oxide/Hydroxyapatite Nanocomposites
Source: Sci Rep. 2015 Dec 21;5:18833. doi: 10.1038/srep18833 (PMC4685392; doi:10.1038/srep18833)
Supplement: Supplementary Information [file srep18833-s1.pdf]

Supporting Information

Enhanced Osteogenesis by Reduced Graphene Oxide/Hydroxyapatite Nanocomposites

Jong Ho Lee[1,+], Yong-Cheol Shin[1,+], Sang-Min Lee[2], Oh Seong Jin[1], Seok Hee Kang[1], Suck Won Hong[1], Chang-Mo Jeong[2], Jung Bo Huh[2,], and Dong-Wook Han[2,**]*

Cytotoxicity assay: MC3T3-E1 preosteoblastic cells were maintained routinely in complete α-Minimum Essential Medium (Sigma-Aldrich Co., St Louis, MO) supplemented with 10% fetal bovine serum (α-MEM, Sigma-Aldrich Co.) and 1% antibiotic antimycotic solution (including 10,000 U penicillin, 10 mg streptomycin, and 25 μg amphotericin B per mL, Sigma-Aldrich Co.) at 37℃ in a humid incubator with 5% CO_2 at 37℃. The number of viable cells was indirectly quantified using a cell counting kit-8 (CCK-8, Dojindo, Kumamoto, Japan), which contains a highly water-soluble tetrazolium salt [WST-8, 2-(2-methoxy-4-nitrophenyl)-3-(4-nitrophenyl)-5-(2,4-disulfophenyl)-2*H*-tetrazolium, monosodium salt] reduced to a yellow-color formazan dye by mitochondrial dehydrogenases. Cell viability was found to be directly proportional to the metabolic reaction products obtained in this assay. Briefly, the CCK-8 assay was conducted as follows: The suspension of MC3T3-E1 cells was seeded at a density of 1×10^5 cells mL^{-1} in a 96-well plate and was then cultured in complete α-MEM at 37℃ under a 5% CO_2 atmosphere until they were grown as monolayer cultures. Cultured cells were treated with increasing concentrations (0 ~ 1,000 μg mL^{-1}) of HAp MPs or increasing concentrations (0 ~ 500 μg mL^{-1}) of rGO NSs and were then incubated with a WST-8 solution for the last 4 h of the culture period (24 h) at 37℃ in the dark. Because residual rGO NSs can affect the absorbance value at 450 nm, cells exposed to rGO NSs were thoroughly washed with 1 × Dulbecco's phosphate-buffered saline (DPBS, Sigma-Aldrich Co.) prior to incubation with a WST-8 solution. Parallel sets of wells containing freshly cultured non-treated cells were regarded as negative (–) controls. The

absorbance was determined at 450 nm using an ELISA reader (SpectraMax® 340, Molecular Device Co., Sunnyvale, CA). Relative cell viability was determined as the percentage ratio of the optical density in the medium (containing HAp MPs or rGO NSs at each concentration) to that in the fresh control medium. The IC_{50}, the concentration (%) inhibiting the growth of cells by 50%, was estimated from relative cell viability profiles.

Cell Culture under Osteogenic Conditions and Osteogenesis Quantification Assays: For osteogenic induction analysis, the suspension of MC3T3-E1 cells was incubated with a colloidal dispersion of HAp MPs (10 μg mL^{-1}), rGO NSs (10 μg mL^{-1}) or rGO/HAp NCs (10 μg mL^{-1}) in osteogenic media [OM, complete α-MEM containing 10 mM β-glycerophosphate, (Sigma-Aldrich Co.), 10 nM dexamethansone (Sigma-Aldrich Co.) and 50 μM L-ascorbic acid (Sigma-Aldrich Co.)] at 37°C in a humidified atmosphere containing 5% CO_2 until they were grown as monolayer cultures. After incubation for 1 to 28 d, the cell proliferation was detected by a CCK-8 assay and osteogenic differentiation was done by an ALP activity assay, ARS staining and Von Kossa staining.

Quantitative Real Time-Polymerase Chain Reaction (qRT-PCR) Analysis: Tissue specimens were fixed in 10% neutral-buffered formalin before being alcohol-dehydrated, cleaned and embedded in paraffin. RNA was extracted from five 10-μm formalin-fixed and paraffin-embedded tissues. Paraffin was removed by xylene extraction followed by ethanol washing. Total RNA was isolated from sectioned tissue blocks using the TRIzol® Reagent (Invitrogen Life Technologies, Carlsbad, CA) according to the supplier's directions. For homogenization, 0.2 mL of chloroform per 1 mL of the reagent was used. Samples were allowed to be incubated for 5 min at room temperature. Samples were centrifuged at 12,000 × *g* for 15 min at 4°C, and the upper phase was transferred to a new tube. The upper phase was mixed with 0.2 mL of isopropanol, and samples were incubated for 10 min at room temperature. Samples were centrifuged at 12,000 × *g* for 15 min at 4°C, and the resulting pellet was washed with 1

mL of 75% ethanol in diethylpyrocarbinate (DEPC)-treated water. After centrifugation at 7,500 × *g* for 10 min, the RNA pellet was air-dried for 5-10 min. The pellets were re-dissolved in 20 μL of DEPC-treated water, allowed to dissolve on ice, and then were ready for reverse transcription. Purified viral total RNA (1 μg) was used as a template to synthesize cDNA by using the TOPscript™ cDNA synthesis kit (Enzynomics, Daejeon, Korea). qRT-PCR reactions were set-up in a total volume of 20 μL with TOPscript™ qPCR 2 × PreMIX (Enzynomics) and 1 μL forward and reverse primers. Primer sequences (5'→3') were as follows: for β-actin: forward, AACTGGAACGGTGAAGGTGA; reverse, CCACATTGCAGAACTTTGGGG; for IL-6 : forward, TGAAGAAGCCACCCTCAAGC ; reverse, TGAAGTGGATCGTGGTCGTC ; for TNF-α : forward, CTTCTCTTTCCTGCTCGTGGC ; reverse, GAGGTTGTTTGGGGACTCTTC. Each sample was run in duplicate in a reaction for 40 cycles using at 94℃ for 15 sec, 53℃ for 15 sec and 72℃ for 15 sec. All PCR reagents were obtained from Applied Biosystems (Foster City, CA).

Micro-CT Analysis: At 4 weeks, harvested calvaria were scanned using a micro-CT scanner (Skyscan 1173; Skyscan, Kontich, Belgium). After calibration, the calvarial specimens were scanned as 0.012 mm-thick sections. Digital micro-radiographic images were acquired at 130 kV and 30 μA. The scanned images were reconstructed using the NRecon software (version 1.6.3.2, Skyscan). The data were analyzed using a CT analyzer (version 1.11.5.1, Skyscan) and remodeled using Realistic 3D-Visualization (Skyscan). The calibrated 3D images were shown in the gross profiles of the specimens. Because the initial defect was round in shape and measuring 6.0 mm in diameter, the setting of the ROI was considered as the initial defect size and shape. The total volume of newly formed bone within the ROI was measured by assigning a threshold for total bone content (including trabecular and cortical bone ranges) and subtracting any contribution from the scaffold.

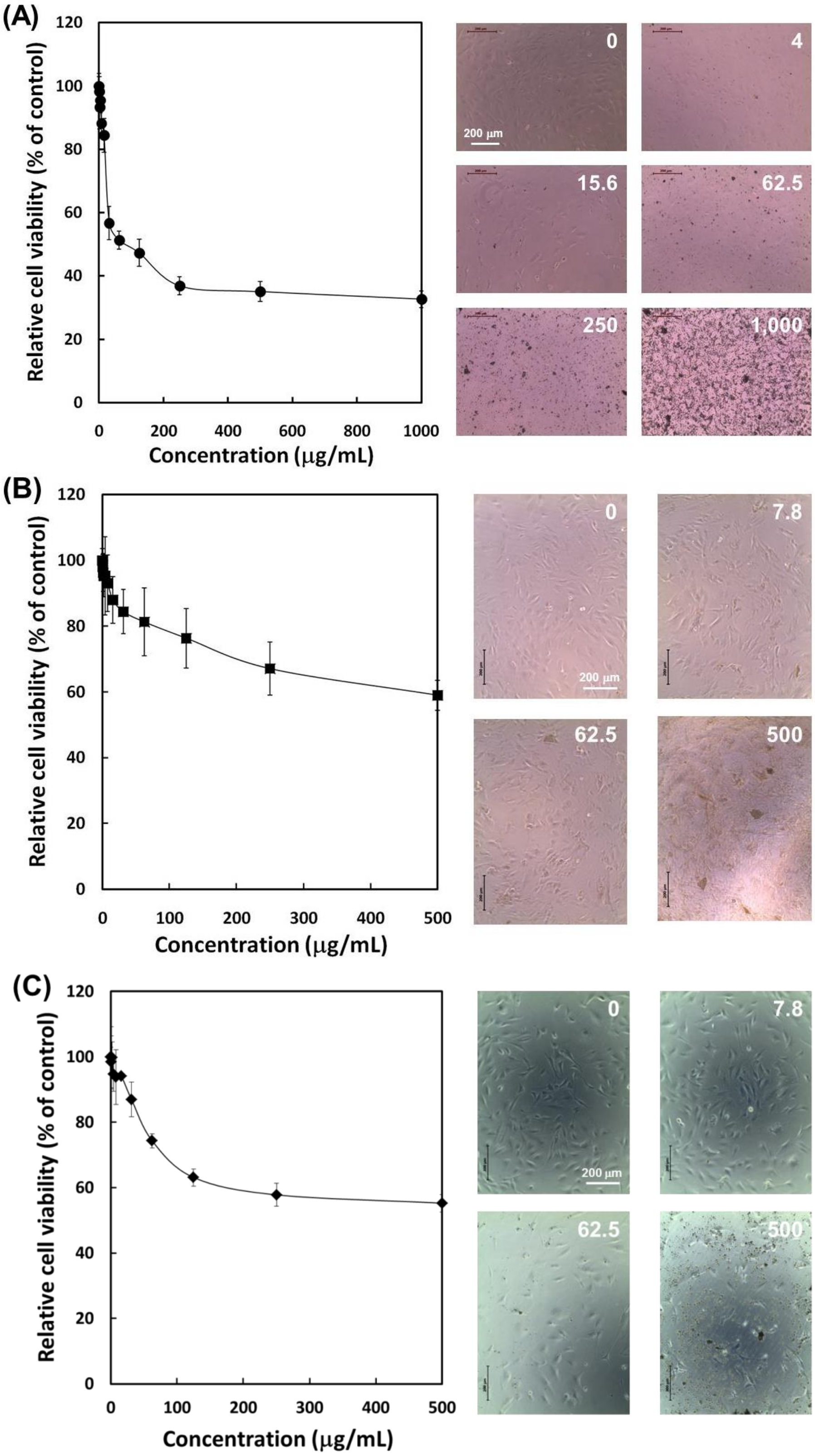

Figure S1. Relative cell viability and morphological alterations of MC3T3-E1 cells exposed to HAp MPs (A), rGO NSs (B) and rGO/HAp NCs with increasing concentrations (0 ~ 500 or 1,000 μg mL^{-1}) for 24 h. All photographs shown in this figure are representative of six independent experiments with similar results.

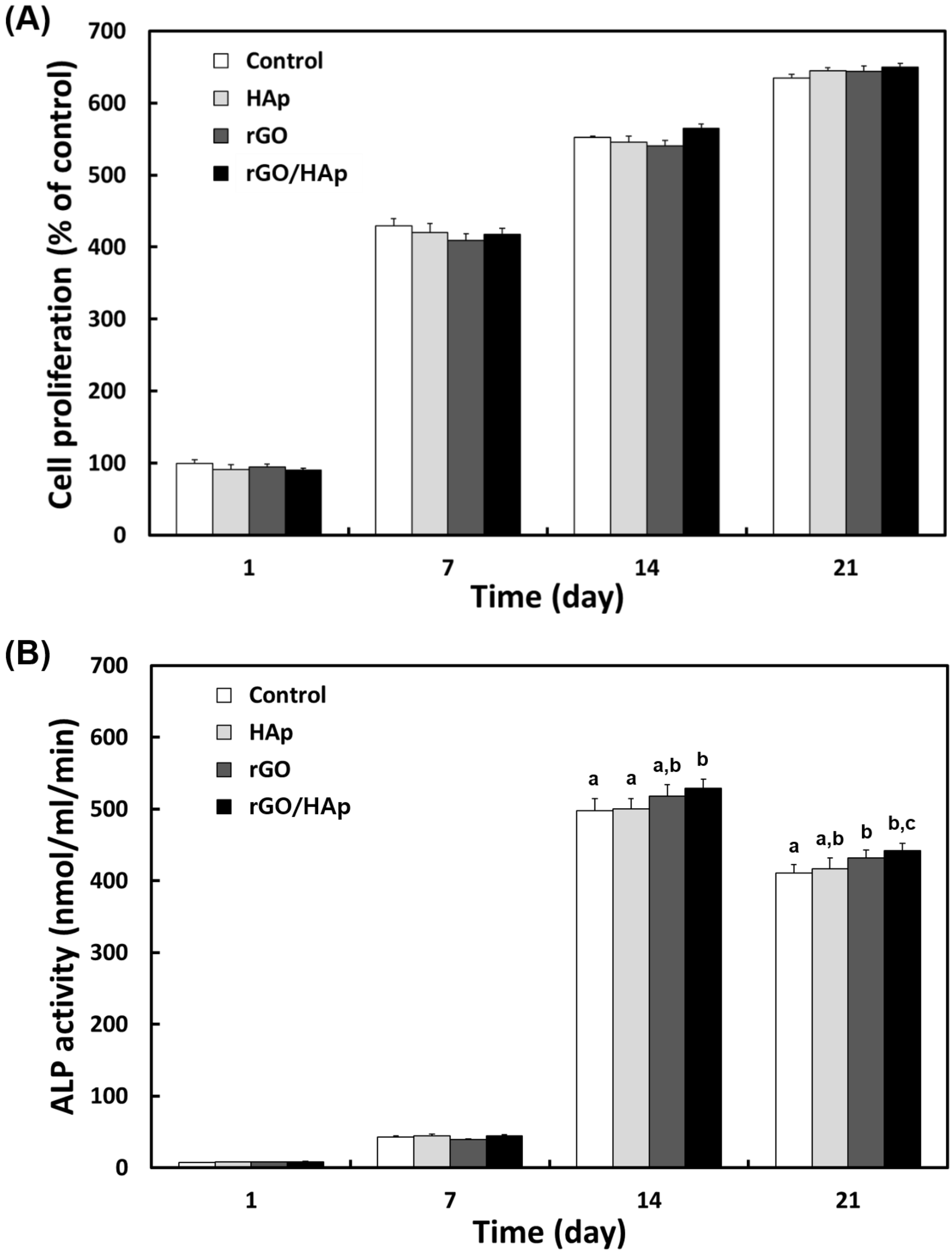

Figure S2. Proliferation and ALP activity of MC3T3-E1 cells incubated with a colloidal dispersion of HAp MPs, rGO NSs or rGO/HAp NCs in OM. (A) During the incubation period (up to 21 d), the cell proliferation pattern was similar to that of cells cultured in basal media (BM) irrespective of the addition of particles or composites. (B) Cells cultured in OM showed remarkably higher ALP activity from 7 d than cells cultured in BM irrespective of the addition of particles. The data is expressed as mean ± SD based on at least duplicate observations from three independent experiments. The different letters in (B) denote significant difference between the non-treated control and cells incubated with particles or composites, $p < 0.05$.

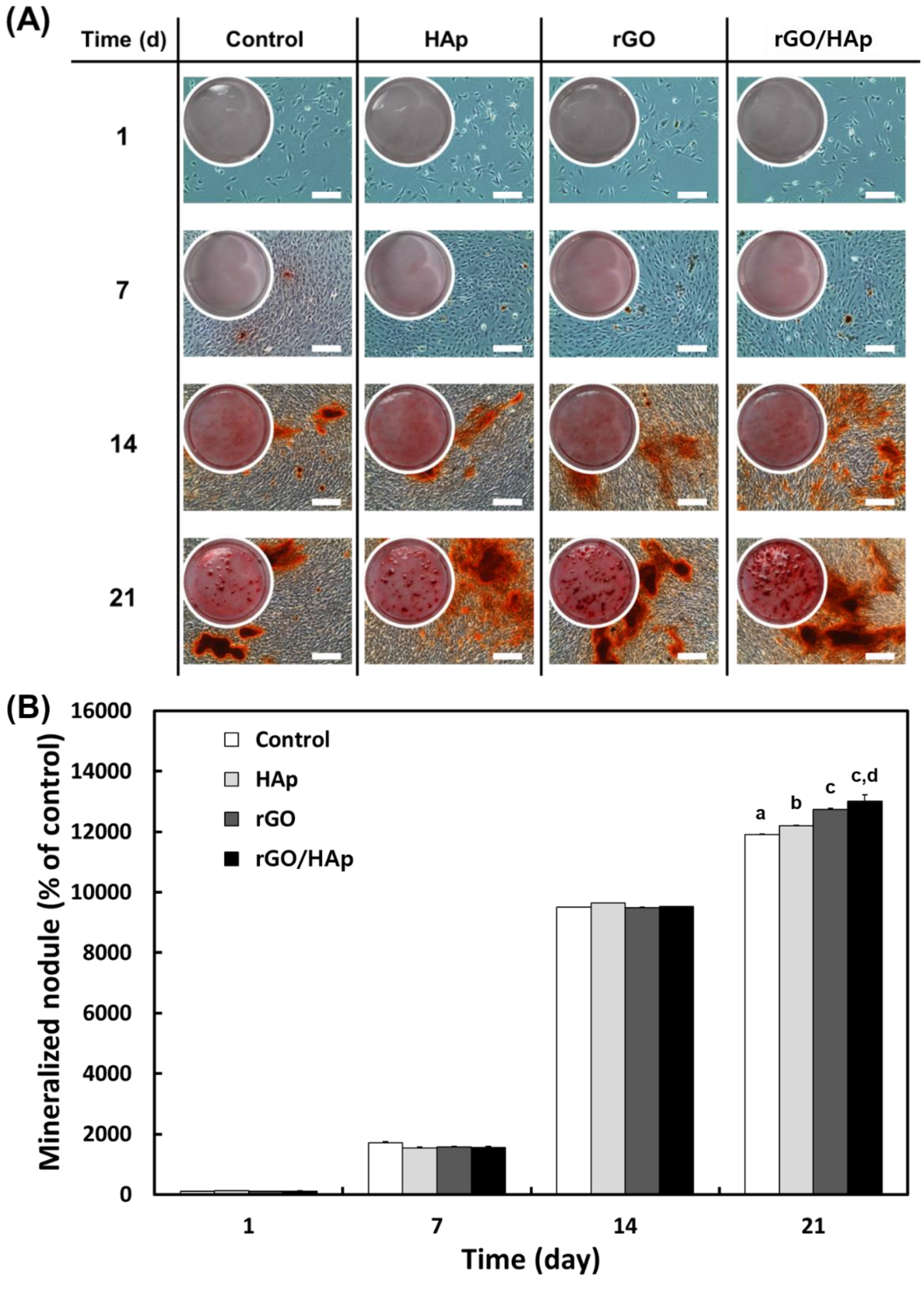

Figure S3. ARS stain and its corresponding extract in MC3T3-E1 cells incubated with a colloidal dispersion of HAp MPs, rGO NSs or rGO/HAp NCs in OM. (A) There was a notable formation of calcium deposits from 14 to 21 d [scale bar = 200 μm]. In contrast to the ARS staining of cells cultured in BM, it shows a diffuse granular appearance of calcium deposits. (B) rGO/HAp NCs significantly ($p < 0.05$) increased extracellular calcium deposition in cells at 21 d. The data is expressed as the mean ± SD based on at least duplicate observations from three independent experiments. The different letters in (B) denote significant difference between the non-treated control and cells incubated with particles or composites, $p < 0.05$. All photographs shown in this figure are representative of six independent experiments with similar results.

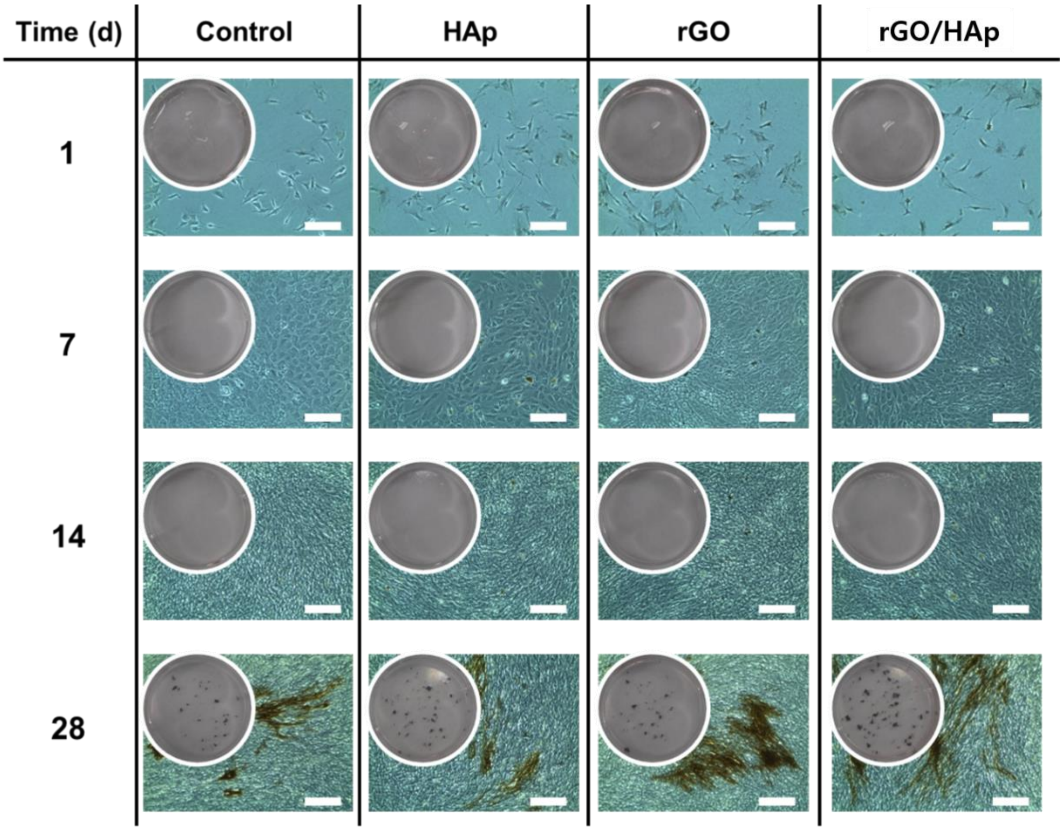

Figure S4. Image of von Kossa stain in MC3T3-E1 cells incubated with a colloidal dispersion of HAp MPs, rGO NSs or rGO/HAp NCs in OM. Dark brown mineralized nodules and crystal formation were observed at 28 d regardless of the addition of particles [a scale bar = 200 μm]. All photographs shown in this figure are representative of six independent experiments with similar results.

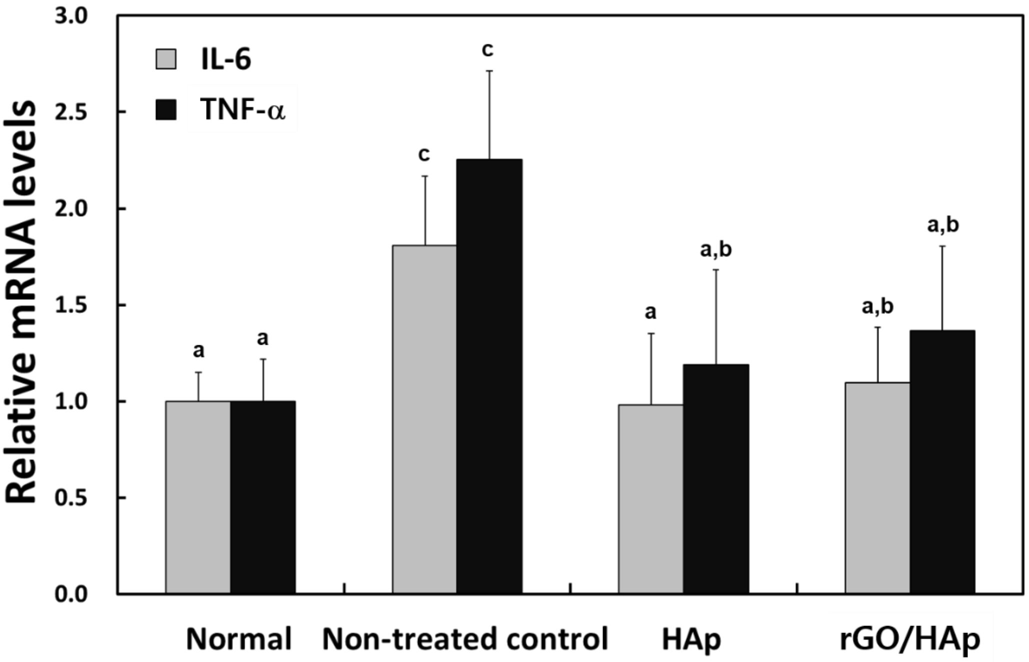

Figure S5. Relative mRNA expression levels of IL-6 and TNF-α in biopsied specimens 4 weeks after implantation of HAp and rGO/HAp grafts into full-thickness bone defects. As compared with the normal tissue, any specific inflammatory responses were not observed in HAp grafts and rGO/HAp grafts at 4 weeks of surgery, but mRNA expression levels of IL-6 and TNF-α in the non-treated control were significantly ($p < 0.05$) increased. The data is expressed as the mean ± SD based on at least duplicate observations from four independent specimens. The different letters denote significant difference between the normal tissue and experimental groups, $p < 0.05$.

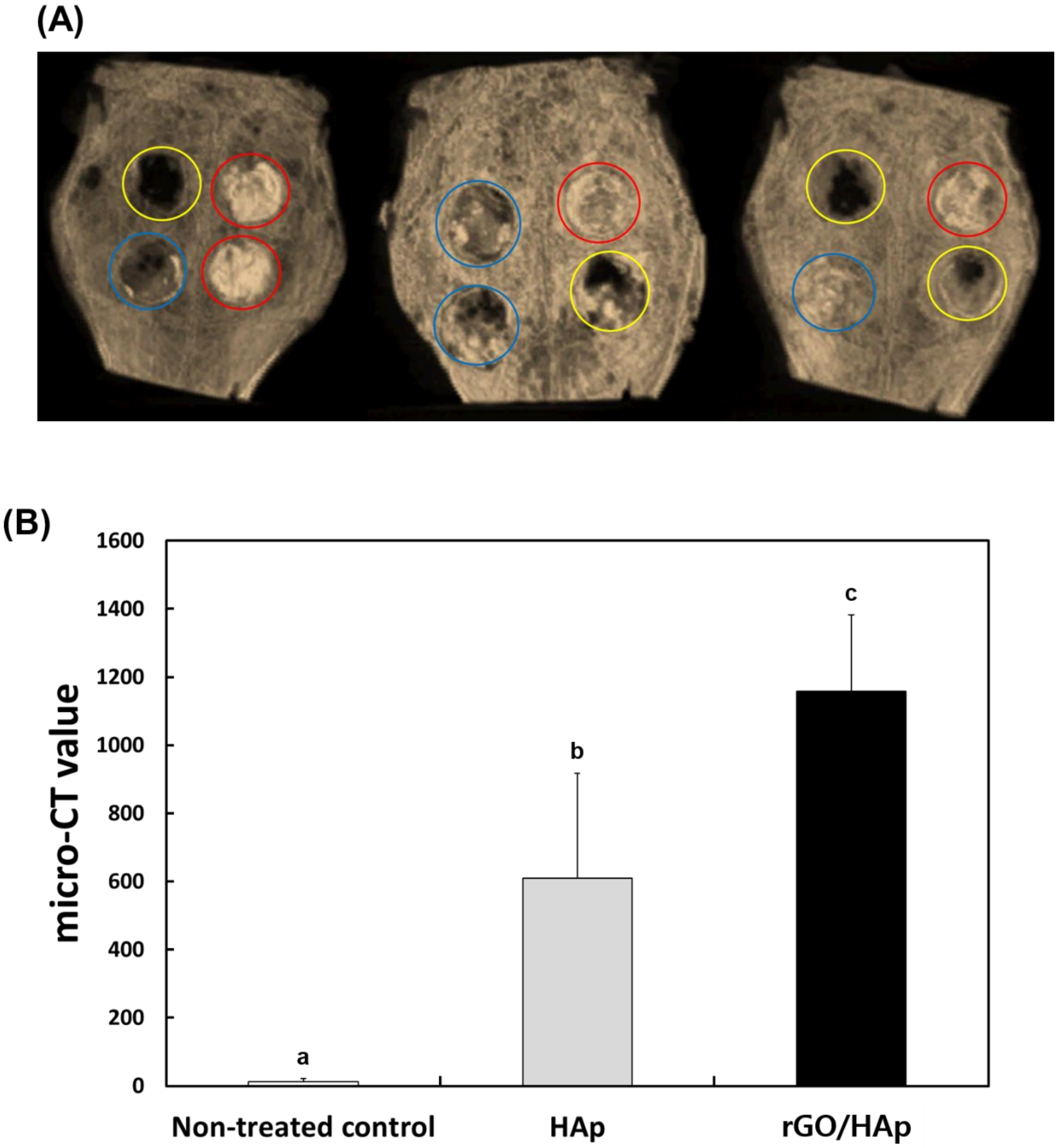

Figure S6. Micro-CT analysis. (A) 3D micro-CT images of experimental groups after 4 week of implantation (Yellow circle: non-treated controls, Blue circle: HAp grafts-implanted and Red circle: rGO/HAp grafts-implanted). Qualitative images revealed that neo-tissue areas formed in both HAp and rGO/HAp grafts substantially greater than that in the non-treated control. (B) Relative micro-CT values for new bone formation. From the quantitative data, it was found that there was significant ($p < 0.05$) difference in the new bone formation between HAp grafts and rGO/HAp grafts. The data is expressed as the mean ± SD based on at least duplicate observations from four independent specimens. The different letters in (B) denote significant difference between the non-treated control and defects implanted with HAp and rGO/HAp grafts, $p < 0.05$.

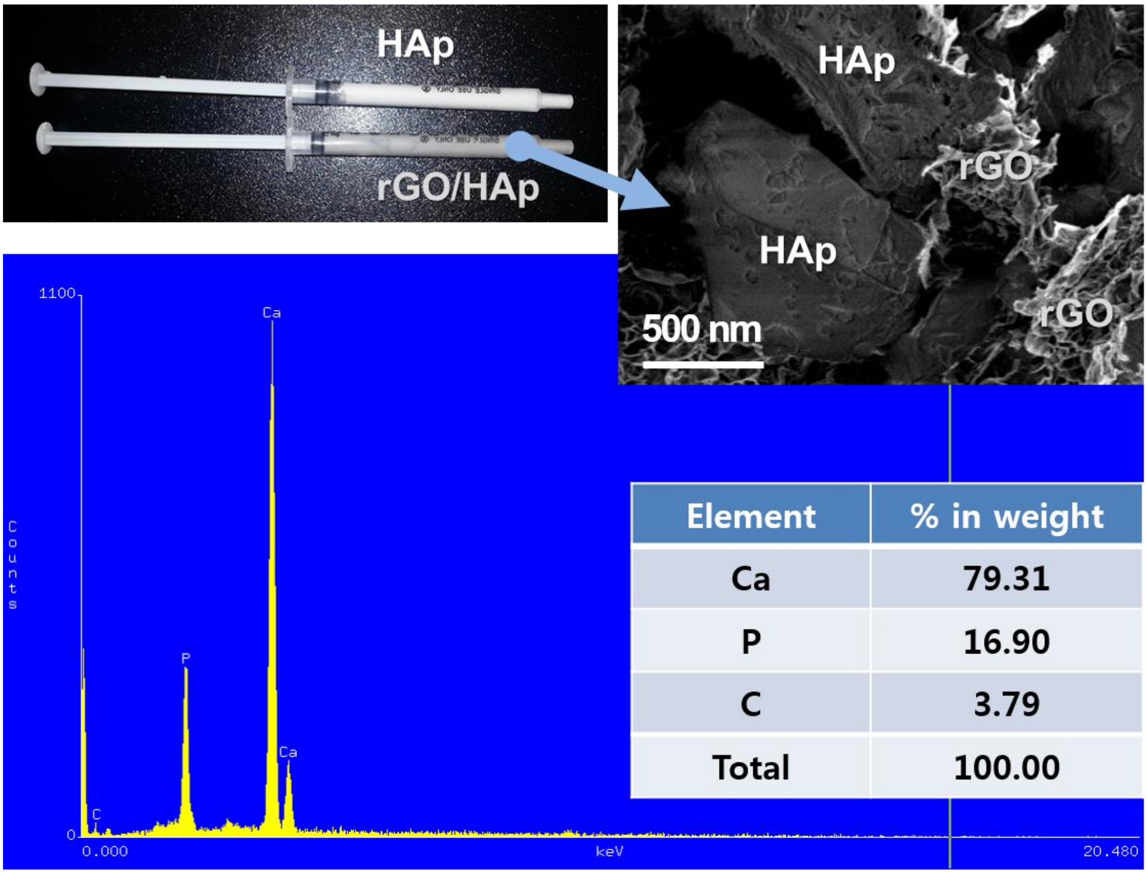

Figure S7. Bone grafts composed of HAp MPs and rGO/HAp NCs prepared in a 1 mL syringe. The SEM micrograph revealed that HAp MPs were partly covered with rGO NSs and the EDX spectrum showed that rGO/HAp grafts were mainly composed of calcium (Ca), phosphor (P), and carbon (C) without any impurities.

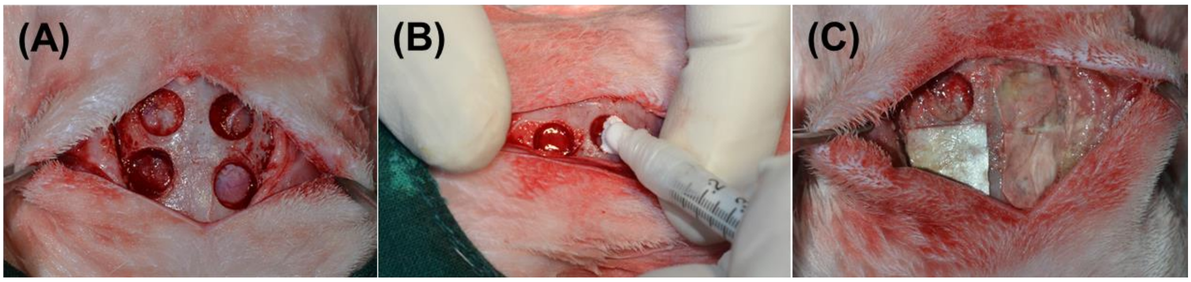

Figure S8. Formation of a full-thickness calvarial defect (6 mm in diameter and 2.5 mm in depth) and the implantation of bone grafts. (A) Four defects were formed using a trephine bur in each calvarium. (B) HAp and rGO/HAp grafts were randomly implanted in defect area. (C) Biocellulose membranes were covered on the top of grafted sites.

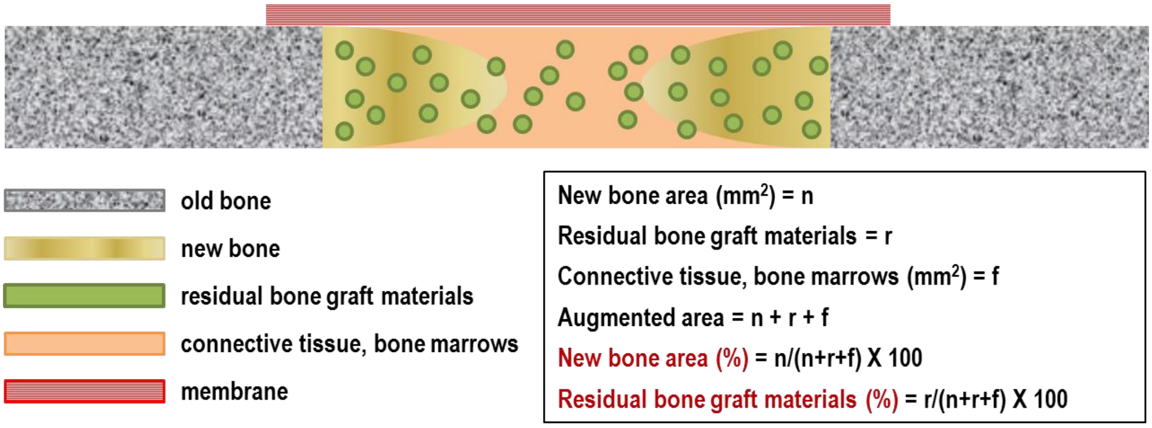

Figure S9. Schematic illustration of calvaria osteotomy defects and histometric analysis with the equation to calculate the percentage ratios of new bone and residual grafts in all defect areas.
